# Assessing Household Solid Fuel Use: Multiple Implications for the Millennium Development Goals

**DOI:** 10.1289/ehp.8603

**Published:** 2006-01-26

**Authors:** Eva Rehfuess, Sumi Mehta, Annette Prüss-Üstün

**Affiliations:** 1 Department of Protection of the Human Environment, World Health Organization, Geneva, Switzerland; 2 Health Effects Institute, Boston, Massachusetts, USA

**Keywords:** child health, environment, indoor air pollution, maternal health, Millennium Development Goals, poverty, solid fuels

## Abstract

**Objective:**

The World Health Organization is the agency responsible for reporting the Millennium Development Goal (MDG) indicator “percentage of population using solid fuels.” In this article, we present the results of a comprehensive assessment of solid fuel use, conducted in 2005, and discuss the implications of our findings in the context of achieving the MDGs.

**Methods:**

For 93 countries, solid fuel use data were compiled from recent national censuses or household surveys. For the 36 countries where no data were available, the indicator was modeled. For 52 upper-middle or high-income countries, the indicator was assumed to be < 5%.

**Results:**

According to our assessment, 52% of the world’s population uses solid fuels. This percentage varies widely between countries and regions, ranging from 77%, 74%, and 74% in Sub-Saharan Africa, Southeast Asia, and the Western Pacific Region, respectively, to 36% in the Eastern Mediterranean Region, 16% in Latin America and the Caribbean and in Central and Eastern Europe. In most industrialized countries, solid fuel use falls to the < 5% mark.

**Discussion:**

Although the “percentage of population using solid fuels” is classified as an indicator to measure progress towards MDG 7, reliance on traditional household energy practices has distinct implications for most of the MDGs, notably MDGs 4 and 5. There is an urgent need for development agendas to recognize the fundamental role that household energy plays in improving child and maternal health and fostering economic and social development.

In September 2000, the largestever gathering of heads of state at the United Nations in New York committed themselves in the Millennium Development Declaration (United Nations 2000) to making the right to development a reality for everyone by the year 2015. The objective of this declaration is to promote a comprehensive approach and a coordinated strategy, tackling many problems simultaneously across a broad front. All 191 member states of the United Nations affirmed that they would “… spare no effort to free our fellow men, women and children from the abject and dehumanizing conditions of extreme poverty, to which more than a billion of them are currently subjected.” (United Nations 2000). To help track progress, a set of eight time-bound and measurable goals for combating poverty, hunger, disease, illiteracy, environmental degradation and discrimination against women were defined (United Nations 2005a):

- Goal 1, eradicate extreme poverty and hunger
- Goal 2, achieve universal primary education
- Goal 3, promote gender equality and empower women
- Goal 4, reduce child mortality
- Goal 5, improve maternal health
- Goal 6, combat HIV/AIDS, malaria and other diseases
- Goal 7, ensure environmental sustainability
- Goal 8, develop a global partnership for development.

Millennium Development Goal (MDG) 7 aims to ensure environmental sustainability, based on the notion that human survival and prosperity critically depend on the sensible use of natural resources and the protection of complex ecosystems. Yet this foundation of our existence is threatened by alarming rates of land degradation and a changing climate. Environmental degradation has disproportionate impacts on the poor, who often rely on the natural resources in their immediate surroundings for their day-to-day subsistence and livelihood.

Worldwide, 2.4 billion people continue to depend on biomass fuels (wood, dung, agricultural residues) to be able to meet their basic energy needs for cooking, boiling water, lighting and, depending on climatic conditions, space-heating [International Energy Agency (IEA) and Organisation for Economic Cooperation and Development (OECD) 2004]. The reliance on wood for fuel can put considerable pressure on forests, particularly in areas where biomass is scarce and the demand for wood outweighs natural regrowth. Depending on the environmental context, deforestation is a driving force for land degradation and desertification. During the 1990s, forest plantations rendered unproductive because of illegal cutting of wood for cooking use were a common sight in China and among the main driving forces for the establishment of the Chinese National Improved Stoves Programme (Energy Sector Management Assistance Programme 1996).

In addition, because biomass fuels are most commonly burnt in open fires or inefficient traditional stoves, a large percentage of the fuel energy is lost as products of incomplete combustion. These include potent greenhouse gases, such as carbon dioxide, methane, and nitrogen dioxide (Edwards et al. 2004; Smith et al. 2000b). In addition, coal use for cooking and heating is still widespread in some countries, China in particular. Consequently, the proportion of the population using solid fuels for cooking is one of the indicators reported to assess progress towards MDG 7.

The World Health Organization (WHO) is the agency responsible for reporting this indicator. In this article, we present the results of the first comprehensive assessment of household solid-fuel use on a country-by-country basis, and we argue that curbing indoor air pollution from solid fuels can make a substantial contribution to reducing child mortality (MDG 4) and improving maternal health (MDG 5). We also discuss the implications of our findings in the context of achieving the MDGs at large.

## Methods

Country data on solid fuel use were estimated for 181 countries. Of these, 93 were compiled from various surveys. The data for 36 countries were modeled based on the proportion of people living in rural areas and the gross national income (GNI) for the year corresponding to the survey data. For 52 upper-middle or high-income countries with a GNI of > US$ 10,500 per capita in 2003, the proportion of population using solid fuels was assumed to be < 5%.

### Indicator definition.

The MDG indicator is defined as the “percentage of population using solid fuels” (United Nations 2005b). Solid fuels include coal, charcoal, wood, crops or other agricultural waste, dung, shrubs, grass, straw, and others (WHO 2005a).

### Data sources.

We compiled data on household solid fuel use from the most recent Demographic and Health Surveys (DHS 2004), the World Health Survey (WHS; WHO 2005b), national censuses, and other sources, such as the World Bank’s Living Standards Measurement Study (LSMS) (World Bank 2006).

DHS surveys are nationally representative household surveys with large sample sizes, ranging from 5,000 to 30,000 households, depending on the size of the country. These surveys are conducted in > 70 countries and are typically repeated every 5 years. Since 1999, the core DHS survey questionnaire asks about the main fuel used for cooking (DHS 2004). The DHS contributed 16 data points to the 2005 assessment.

The WHS, a nationally representative survey with sample sizes of between 1,000 and 10,000 households, was carried out in 2003–2004, and 50 countries collected information on the main fuel used for cooking (WHO 2005b). The 49 WHS data points used in this assessment are early release data and final estimates may change from those reported here.

### Modeling.

A statistical model was used to predict household solid fuel use for those countries where no data were available. This model was originally developed to obtain global and regional estimates of solid fuel use in the context of WHO’s comparative risk assessment (CRA) (Smith et al. 2004; WHO 2002). Model parameters were defined by explaining data on household solid fuel use obtained from surveys. The model was then applied to countries for which no household solid fuel–use data were available.

The model was built using stepwise linear regression and testing a wide range of parameters. Missing values were replaced with mean values for each variable. The following variables were not significant: petroleum use per capita, adult female literacy, average annual growth rate, dummy variables for all WHO Regions (with the exception of the Eastern Mediterranean Region), electricity consumption per capita, fuel wood production, and traditional fuel use at the national level (vs. at the household level). The final model parameters were identified as a country’s gross national income (GNI), percentage of the rural population, and location or nonlocation within the Eastern Mediterranean region.

An *R*^2^ value of 0.78 suggests that the model performs reasonably well. With a beta coefficient of 0.891 [95% confidence interval (CI), 0.651−1.131), the percentage of the rural population appears to have the highest explanatory power, followed by GNI (−0.184; 95% CI, −0.237 to −0.131) and location within the Eastern Mediterranean region (−0.169; 95% CI, −0.280 to −0.058). The latter is likely to be important because several of the countries in this region have substantial oil and gas resources. We used SPSS, version 8.0 (SPSS, Chicago, IL, USA) and STATA 7.0 (StataCorp, College Station, TX, USA) for analyses. The model has been described in detail elsewhere (Mehta et al., in press; Smith et al. 2004).

## Results

The resulting country data for percentage of population using solid fuels are listed in Table 1. These represent the most recent data available for all developing and industrializing countries, dating from 1990 to 2004. Data for industrialized countries, as well as years for individual countries, are available from the website of the United Nations Statistics Division (United Nations 2005b).

More than half of the world’s population still uses solid fuels for cooking. Reliance on solid fuels varies widely between different world regions. In Sub-Saharan Africa, Southeast Asia, and the Western Pacific Region, the use of solid fuels prevails over cleaner fuel options, reaching 77%, 74%, and 74%, respectively. In the Eastern Mediterranean Region, traditional household energy practices are also significant, with 36% of the population using solid fuels for cooking. With only 16% prevalence, the use of solid fuels is less common in Latin America and the Caribbean and in Central and Eastern Europe.

## Discussion

### Trends and changes.

Most national censuses, energy assessments, or household surveys have only recently integrated questions relating to solid fuel use. It is thus impossible to draw firm conclusions on trends in household energy use over the past decade.

We have, however, observed differences in the 2005 assessment (using data up to the year 2003) compared with the 2002 assessment of solid fuel use ( using data up to the year 2000) (Smith et al. 2004). The 2002 assessment revealed that 57% of the world’s population relied on solid fuels, whereas our recent estimates indicate that, globally, 52% of the population cook with solid fuels. In fact, the MDG indicator has seen a small decrease for most of the WHO epidemiologic subregions since the 2002 assessment (Smith et al. 2004) for example, from 73% to 70% in African countries with high child and adult mortality, 53% to 43% in countries of the Americas with high child and adult mortality, and 78% to 74% in countries of the Western Pacific Region with low child and high adult mortality. In selected countries and WHO regions, these changes may signal real improvements. In most cases, however, we believe that improvements in data availability and estimation methods are the main cause for the observed changes. The 2002 assessment (Smith et al. 2004) compiled data from surveys for only 52 countries, requiring the majority of data points to be modeled. In contrast, the 2005 assessment is based on survey data for 93 countries; therefore, solid fuel use was modeled for only 36 countries.

### Implications for achieving MDGs 4 and 5.

Three out of the eight MDGs directly address health (MDGs 4, 5, and 6). The widespread use of solid fuels to meet basic energy needs represents a major public health concern with important implications for MDGs 4 and 5.

Cooking and heating with solid fuels on open fires or traditional stoves in poorly ventilated indoor environments leads to high levels of indoor air pollution. Indoor air pollution comprises a variety of health-damaging pollutants including particles, carbon monoxide, nitrous oxides, sulfur oxides (mainly from coal), formaldehyde, and carcinogens, such as benzo[*a*]pyrene and benzene (Smith 1987). These pollutants mainly affect the lungs by causing inflammation, reduced ciliary clearance, and impaired immune response (Bruce et al. 2000). CO also results in systemic effects by reducing the oxygen-carrying capacity of the blood (Boy et al. 2002). Small particles, including particles with a diameter of ≤10 μm (PM10), respirable particles (PM3.5), and particles with a diameter of ≤2.5 μm (PM2.5), are able to penetrate deep into the lungs and appear to have the greatest health-damaging potential (Bruce et al., in press).

Studies from Asia, Africa, and the Americas have shown that indoor air pollution levels are extremely high in households that rely on biomass fuel or coal: for example, 24-hr mean levels for PM10 in homes using biomass fuels range from 300 to 3,000 μg/m^3^ (WHO 2005c). By comparison, the U.S. Environmental Protection Agency (EPA) annual air pollution standard for PM10 is 50 μg/m^3^ (U.S. EPA 1996). Thus, typical concentrations of indoor air pollutants exceed many times the generally accepted guideline limits for outdoor air pollution. Even in households using more fuel-efficient and less-polluting improved stoves, typical concentrations often exceed such guideline limits. For example, in a recent evaluation of the Chinese National Improved Stoves Programme, Sinton et al. (2004) found PM4 (the thoracic fraction of particulate matter) higher than the Chinese national indoor air standard of 150 μg/m^3^ for PM10 in homes using improved biomass stoves; if their evaluation had measured PM10,exceedance of the standard would have been even more pronounced. A person’s exposure to indoor air pollution is determined by the concentration of pollutants in the indoor environment and by the amount of time spent in this environment. Few studies have assessed personal exposure of women or children to small particles or CO, but Ezzati and Kammen (2001) found 24-hr exposures to PM10 from cooking range from several hundred micrograms per cubic meter to > 1,000 μg/m^3^. These findings imply that most of the 3.2 billion people using solid fuels for cooking (United Nations 2005b) are exposed to levels of indoor air pollution that exceed accepted guideline levels for outdoor air pollution on a daily basis with dramatic consequences for health.

Indoor air pollution has been associated with a wide range of health outcomes, and the evidence for these associations was classified as strong, moderate, or tentative in a recent systematic review (Smith et al. 2004). There is consistent evidence that exposure to indoor air pollution increases the risk of pneumonia and other acute lower respiratory infections (ALRIs) among children under 5 years of age (Bruce et al. 2000; Collings et al. 1990; de Francisco et al. 1993; Johnson and Aderele 1992; O’Dempsey et al. 1996; Pandey et al. 1989; Robin et al. 1996; Smith et al. 2000a), chronic obstructive pulmonary disease (COPD) (Dennis et al. 1996; Pandey 1984; Perez-Padilla et al. 1996), and lung cancer (in relation to coal use) among adults above 30 years of age (Dai et al. 1996; Liu et al. 1993; Wang et al. 1996; Wu-Williams et al. 1990). The evidence for a link with lung cancer from exposure to biomass smoke—and for a link with asthma, cataracts, and tuberculosis—was considered moderate. Given limited available studies to date, there is tentative evidence for an association between indoor air pollution and adverse pregnancy outcomes, in particular low birth weight, ischaemic heart disease, interstitial lung disease, and nasopharyngeal and laryngeal cancers.

The WHO, through the CRA, estimated the contribution of a range of risk factors to the global and regional burden of disease (WHO 2002). With respect to indoor air pollution, the assessment included only ALRI, COPD, and lung cancer (from coal use)—those health outcomes for which the evidence for indoor air pollution as a cause was classified as strong. Indoor air pollution was identified as the eighth most important risk factor and responsible for 2.7% of the global burden of disease. Globally, indoor air pollution from solid fuel use accounted for 1.6 million deaths and 39 million disability-adjusted life years (DALYs; a measure combining years of life lost due to disability and death) in the year 2000 (Smith et al. 2004). In developing countries with high-mortality, indoor air pollution was responsible for 3.7% of the overall disease burden, making it the most important risk factor after malnutrition, unsafe sex, and lack of safe water and adequate sanitation (WHO 2002).

The 2005 assessment indicates that global solid fuel use is slightly lower (52%) than estimated during the 2002 assessment (57%) (Smith et al. 2004) that formed the basis for estimating the burden of disease attributable to indoor air pollution from solid fuel use. However, when looking at absolute numbers that incorporate population growth, the differences between the 2002 and 2005 assessments become less substantial with 3.4 billion people in 2000 and 3.2 billion people in 2003 using solid fuels. This implies that the CRA may have slightly overestimated the global burden of disease caused by indoor air pollution-attributable ALRI, COPD, and lung cancer. On the other hand, the CRA followed a very conservative approach by including only those health outcomes for which the evidence was considered strong. We can expect that the global burden of disease caused by indoor air pollution will exceed the originally estimated 1.6 million deaths and 39 million DALYs as the evidence base for links between indoor air pollution and additional health outcomes (e.g., low birth weight, tuberculosis, and cataract) increases through new studies. For example, in a recent Nepalese study, Pokhrel et al. (2005) confirmed the findings of previous studies in identifying indoor air pollution as a risk factor for cataract.

#### MDG 4 strives to reduce mortality in children < 5 years of age.

**Figure 1:**
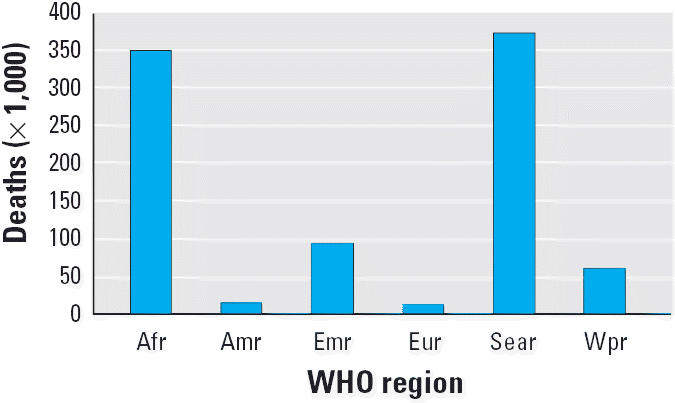
Geographic distribution of ALRI deaths attributable to indoor air pollution in children < 5 years of age, by WHO region in 2000 (based on data from Smith et al. 2004). Abbreviations: Afr, African Region; Amr, Region of the Americas; Emr, Eastern-Mediterranean Region; Eur, European Region; Sear, Southeast Asian Region; Wpr, Western Pacific Region.

Globally, pneumonia represents the single most important cause of death in children under 5 years of age and is responsible for approximately 2 million deaths every year (WHO 2005d). Young children are often carried on their mother’s back during cooking or kept close to the warm hearth. Consequently, children spend many hours breathing indoor air pollution during their first year of life, when their developing airways and immature immune systems make them particularly vulnerable to hazardous pollutants. In their meta-analysis, Smith et al. (2004) found a relative ALRI risk of 2.3 (95% CI, 1.9–2.7) among children exposed to indoor air pollution. Consequently, indoor air pollution is responsible for > 900,000 annual deaths (56% of all indoor air pollution–attributable deaths) due to ALRI in children < 5 years of age. As illustrated in Figure 1, these deaths are not equally distributed across the globe: More than one-third of all child deaths caused by indoor air pollution (i.e. 350,000 deaths) occur on the African continent, and another 374,000 occur in Southeast Asia (Smith et al. 2004; WHO 2005e).

Most users of solid fuels are poor and, especially in rural areas, are unlikely to live in the vicinity of primary health care facilities or hospitals; their ability to afford medical treatment and seek medical care for their sick children at an early stage is limited. Consequently, reducing pneumonia deaths through treatment may not reach the poorest of the poor. Even if a child is successfully treated for pneumonia, he or she will return to a home where high levels of indoor air pollution prevail in combination with other risk factors for pneumonia, such as overcrowding and an inadequate diet. In contrast, reducing exposure to indoor air pollution can reduce the risk of pneumonia for all children in the household on a long-term basis and thus make a significant contribution to reducing child morbidity and mortality.

Improvements in household energy practices, in particular, the use of improved stoves or the use of cleaner fuels, can bring about additional benefits to children’s health. Exposure of the developing embryo to indoor air pollution may contribute to perinatal mortality and low birth weight, a major risk factor for a variety of diseases during childhood (Boy et al. 2002; Bruce et al. 2000; Mavlankar et al. 1991; Mishra et al. 2004, 2005). Improved access to household energy can facilitate boiling of water and thus help reduce the incidence of waterborne diseases (United Nations Millennium Project 2005); it can also increase the number of hot meals per day and thus improve food safety. Moreover, among infants and toddlers, open fires in the kitchen and kerosene lamps are a major cause of burns and scalds; as a result of children falling into open fires or knocking over pots of hot liquid or kerosene lamps (Courtright et al. 1993; Onuba and Udoidiok 1987).

#### MDG 5 aims to improve maternal health.

In most societies, women are in charge of cooking and thus are more at risk of the health impacts of indoor air pollution than men. Women exposed to indoor smoke are 3.2 times (95% CI, 2.3–4.8) more likely to suffer from COPD, such as chronic bronchitis and emphysema, than women who cook and heat with electricity, gas, or other cleaner fuels. Indoor air pollution is responsible for 522,000 of the 1.3 million global deaths from COPD among women (independent of the effects of smoking). In contrast, only 171,000 of a total of 1.4 million COPD deaths among men are attributable to indoor air pollution exposure (Smith et al. 2004; WHO 2005e).

Tobacco smoking is the principal risk factor for lung cancer worldwide, but smoking rates among women in most developing countries tend to be very low. In China and some Central Asian countries, exposure to indoor air pollution from coal fires is associated with a 1.9-fold increased risk of lung cancer among women (95% CI, 1.1–3.5). With 9,000 annual deaths attributable to indoor air pollution due to lung cancer, bronchus cancer, and tracheal cancer among women, the Western Pacific Region is the most affected.

**Figure 2:**
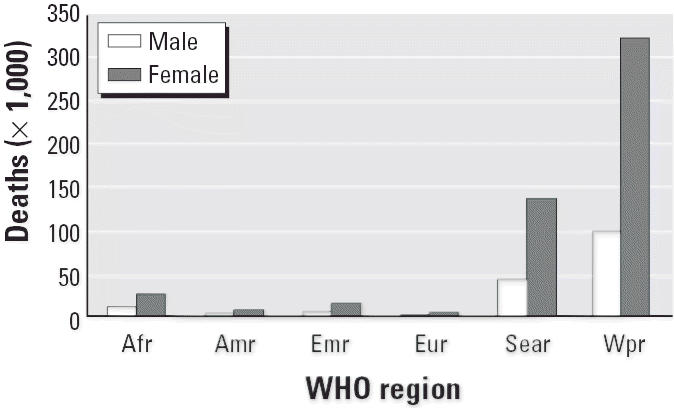
Geographic distribution of COPD deaths attributable to indoor air pollution, by sex and WHO region in 2000 (based on data from Smith et al. 2004). Abbreviations: Afr, African Region; Amr, Region of the Americas; Emr, Eastern-Mediterranean Region; Eur, European Region; Sear, Southeast Asian Region; Wpr, Western Pacific Region.

Curbing indoor air pollution can thus make an important contribution to improving respiratory health among women, in particular, among young mothers who, in many societies, tend to spend much time close to the warm hearth after giving birth. In Asia alone, > 460,000 deaths due to chronic respiratory disease among women could be prevented every year (Figure 2). Easier access to other fuels and higher fuel efficiency are likely to bring about additional health improvements to pregnant women and mothers. For example, some studies suggest that carrying heavy loads (e.g., biomass fuels) may be associated with an increased risk of prolapse (Pandey 1997).

### *Implications for achieving* MDGs *1 and 3*.

Going beyond health, the fact that half of the world’s population relies on solid fuels has many consequences for economic development.

#### MDG 1 to eradicate extreme poverty and hunger represents the essence of the Millennium Declaration and plays an overarching role for achieving the MDGs overall.

The security of household livelihoods rests on the health of its members. Being ill as a result of exposure to indoor air pollution (MDG 5) or having to care for sick children (MDG 4) reduces earning capacities and leads to additional expenses for health care and medication.

One of the targets established to measure progress towards achieving MDG 1 is halving the proportion of the world’s people living on < $ 1 (purchasing power parities)/day by 2015. Based on the strong link between income and access to energy services, whether at the global or national level, the IEA and OECD (2004) predicted that this goal can only be met if “governments act decisively to accelerate the transition to modern fuels and to break the vicious cycle of energy poverty and human underdevelopment in the world’s poorest countries.” More specifically, to achieve the poverty-reduction target, the number of people relying almost entirely on traditional biomass for cooking and heating must be reduced to < 1.85 billion. Yet, according to the IEA’s reference scenario (IEA and OECD 2004), this number will increase from 2.40 billion in 2002 to 2.55 billion in 2015 and, even further, to 2.63 billion in 2030. Therefore, to accomplish the poverty-reduction target in the light of population growth, governments will need to extend the use of modern cooking and heating fuels to an additional 700 million people by 2015.

#### MDG 3 is dedicated to women.

We have dicsussed the unequal impacts of exposure to indoor air pollution on women’s health in relation to MDG 5, yet reliance on traditional cooking and heating practices has additional implications for female household members.

Women and children tend to be in charge of fuel collection in those areas where household energy needs are met through fuel collection rather than purchase. The Energy Sector Management Assistance Programme (2004) revealed that women cooks in rural India spend an average of 40 min/day on fuel collection in addition to an average 3 hr/day spent on cooking and serving food. Collection time varies depending on local fuel wood availability. A study in rural Malawi demonstrated that women, assisted by young girls, spent between 4 hr (short distance from woodland) and 15 hr (long distance from woodland) per week on fuel collection (Brouwer 1998). In rural India, daily fuel collection time ranged from only 20 min/day in Andhra Pradesh to more than 1 hr/day in Rajasthan, which is mostly covered by desert (Energy Sector Management Assistance Programme 2004). To date, there is no systematic quantitative assessment of injury (e.g., due to backache, broken bones, or snake bites) or assault endured during fuel collection, yet girls and women are at particular risk, even more so in war zones or refugee camps (Intermediate Technology Development Group 2004; United Nations High Commissioner for Refugees 2001).

Alleviating the drudgery of fuel collection far from home and reducing cooking time through more efficient devices can free women’s time for productive endeavours (MDG 1), adult education, and child care, and will reduce the risk of assault and injury for women and girls. Moreover, involving women in household energy decisions contributes to promoting gender equality and empowering women. Owning a less-polluting stove can raise a woman’s prestige, both as a sign of wealth and, indirectly, through a soot-free kitchen environment.

## Outlook

What does the current indicator actually tell us? From an environmental point of view, widespread use of solid fuels could mistakenly be interpreted as a positive development, given that most biomass fuels—the major share of solid fuels—constitute a source of renewable energy. From a public health point of view, widespread use of solid fuels is, of course, interpreted as a negative development because of the health risks associated with indoor air pollution. Yet, solid fuel use is a poor proxy for indoor air pollution levels as the concentrations of small particles, CO, and other pollutants vary markedly between different types of solid fuels and between the same fuel being burnt in a traditional open fire versus in a well-maintained improved stove. In light of population growth and given the current lack of political commitment, it seems unlikely that the coming decade will witness a large reduction in solid fuel use. In most of the developing world, improved stoves are likely to remain the most feasible and cost-effective intervention to reduce indoor air pollution in the short-term (Mehta and Shapar 2004), and the current indicator alone will not allow us to demonstrate such progress.

To improve accuracy in estimating health and other impacts and to provide a better basis for designing interventions, current limitations in the definition and reporting of the indicator should be overcome. To date, lack of comparable data on a country-by-country basis has prevented us from reporting household energy practices in more detail. Efforts are under way to improve regular data collection through international surveys, such as the DHS, the LSMS and UNICEF’s Multiple Indicator Cluster Survey by integrating additional questions on type of cooking stove and kitchen characteristics within the harmonized questionnaires. Furthermore, given stark differences between urban and rural populations, we propose to disaggregate household energy practices according to place of residence.

By drawing on the classification of “the proportion of the population relying on solid fuels” as an indicator to measure progress towards achieving MDG 7, we have outlined how exposure to indoor air pollution from solid fuels has distinct implications for achieving MDG 4 to reduce child mortality and MDG 5 to improve maternal health. Moreover, overcoming energy poverty will play a key role in attaining MDG 1 to eradicate extreme poverty and hunger and MDG 3 to promote gender equality and empower women. Development agendas and partnerships must recognize the fundamental role that household energy plays in improving child and maternal health and in fostering economic and social development.

An important step in this direction is the recent report *Investing in Development: A Practical Plan to Achieve the Millennium Development Goals* (United Nations Millennium Project 2005). The report recommends that countries adopt the specific target to reduce the number of people without effective access to modern cooking fuels by 50% and make improved cookstoves widely available by 2015. Can this ambitious goal be achieved over the course of the coming decade? Success stories, such as the Chinese National Improved Cookstoves Programme and the Brazilian efforts to increase use of liquid petroleum gas and other modern fuels (Modi et al. 2005), indicate that rapid progress is possible. Yet, the world will need to embrace safe cooking fuels and other essential energy services as “… vital to enabling and facilitating the achievement of the Millennium Development Goals” (United Nations Millennium Project 2005). With a sense of urgency, we must act in concert to make the necessary policy changes and implement technical solutions that can improve the lives of > 3 billion fellow humans.

## Figures and Tables

**Table 1:** Percentage of population using solid fuels, by country and WHO region.

Region/country	Percentage
Africa	77
Algeria	< 5
Angola	> 95
Benin	95
Botswana	65
Burkina Faso	> 95
Burundi	> 95
Cameroon	83
Cape Verde	36
Central African Republic	> 95
Chad	> 95
Comoros	76
Congo	84
Cote d'Ivoire	74
Democratic Republic of the Congo	> 95
Equatorial Guinea	ND
Eritrea	80
Ethiopia	> 95
Gabon	28
Gambia	> 95
Ghana	88
Guinea	> 95
Guinea-Bissau	95
Kenya	81
Lesotho	83
Liberia	ND
Madagascar	> 95
Malawi	> 95
Mali	> 95
Mauritania	65
Mauritius	< 5
Mozambique	80
Namibia	63
Niger	> 95
Nigeria	67
Rwanda	> 95
Sao Tome and Principe	ND
Senegal	41
Seychelles	< 5
Sierra Leone	92
South Africa	18
Swaziland	68
Togo	76
United Republic of Tanzania	> 95
Uganda	> 95
Zambia	85
Zimbabwe	73
Latin America and the Caribbean	16
Antigua and Barbuda	46
Argentina	< 5
Bahamas	< 5
Barbados	< 5
Belize	43
Bolivia	25
Brazil	12
Chile	< 5
Colombia	15
Costa Rica	23
Cuba	< 5
Dominican Republic	14
Ecuador	< 5
El Salvador	33
Grenada	48
Guatemala	62
Guyana	59
Haiti	> 95
Honduras	57
Jamaica	45
Mexico	12
Nicaragua	58
Panama	33
Paraguay	58
Peru	33
Saint Kitts and Nevis	< 5
Saint Lucia	63
St Vincent and the Grenadines	31
Suriname	ND
Trinidad and Tobago	8
Uruguay	< 5
Venezuela	5
Eastern Mediterranean	36
Afghanistan	> 95
Bahrain	< 5
Cyprus	< 5
Djibouti	6
Egypt	< 5
Iran, Islamic Republic of	< 5
Iraq	< 5
Jordan	< 5
Kuwait	< 5
Lebanon	< 5
Libyan Arab Jamahiriya	< 5
Morocco	5
Oman	< 5
Pakistan	72
Qatar	< 5
Saudi Arabia	< 5
Somalia	ND
Sudan	> 95
Syrian Arab Republic	32
Tunisia	5
United Arab Emirates	< 5
Yemen	42
Central and Eastern Europe	16
Albania	50
Armenia	26
Azerbaijan	49
Belarus	19
Bosnia and Herzegovina	51
Bulgaria	17
Estonia	15
Georgia	42
Hungary	< 5
Kazakhstan	5
Kyrgyzstan	76
Latvia	10
Lithuania	< 5
Poland	< 5
Republic of Moldova	63
Romania	23
Serbia and Montenegro	ND
Slovakia	< 5
Tajikistan	75
TFYR of Macedonia	30
Turkey	11
Turkmenistan	< 5
Ukraine	6
Uzbekistan	72
Russian Federation	7
Southeast Asia	74
Indonesia	72
Sri Lanka	67
Thailand	72
Bangladesh	88
Bhutan	ND
India	74
Korea, Democratic People's Republic of	ND
Maldives	ND
Myanmar	95
Nepal	80
Timor-Leste	ND
Western Pacific	74
Cambodia	> 95
China	80
Cook Islands	ND
Fiji	40
Kiribati	ND
Korea, Republic of	< 5
Lao People's Democratic Republic	> 95
Malaysia	< 5
Marshall Islands	ND
Micronesia, Federated States of	ND
Mongolia	51
Nauru	ND
Niue	ND
Palau	ND
Papua New Guinea	90
Philippines	47
Samoa	70
Singapore	< 5
Solomon Islands	95
Tonga	56
Tuvalu	ND
Vanuatu	79
Viet Nam	70
World	52

ND, no data. Data from United Nations (2005b). For a more detailed explanation of WHO regions and epidemiologic subregions based on mortality strata, see WHO (2002).

## References

[b1-ehp0114-000373] Boy E, Bruce N, Delgado H (2002). Birth weight and exposure to kitchen wood smoke during pregnancy. Environ Health Perspect.

[b2-ehp0114-000373] Brouwer ID 1998. When households run out of fuel: responses of rural women in Malawi to decreasing fuelwood availability. Energia News 2(2). Available: http://www. energia.org/resources/newsletter/en_051998_artib.html [accessed 31 January 2006].

[b3-ehp0114-000373] Bruce NG, Perez-Padilla R, Albalak R (2000). Indoor air pollution in developing countries: a major environmental and public health challenge. Bull WHO.

[b4-ehp0114-000373] Bruce N Rehfuess E Mehta S Hutton G Smith KR In press. Indoor air pollution. In: Disease Control Priorities in Developing Countries. 2nd ed. Washington, DC/Oxford, UK:World Bank/Oxford University Press.

[b5-ehp0114-000373] Collings DA, Sithole SD, Martin KS (1990). Indoor woodsmoke pollution causing lower respiratory disease in children. Trop Doct.

[b6-ehp0114-000373] Courtright P, Haile D, Kohls E (1993). The epidemiology of burns in rural Ethiopia. J Epidemiol Community Health.

[b7-ehp0114-000373] Dai X, Lin CY, Sun XW, Shi YB, Lin YJ (1996). The etiology of lung cancer in nonsmoking females in Harbin, China. Lung Cancer.

[b8-ehp0114-000373] de Francisco A, Morris J, Hall AJ, Armstrong Schellenberg JR, Greenwood BM (1993). Risk factors for mortality from acute lower respiratory tract infections in young Gambian children. Int J Epidemiol.

[b9-ehp0114-000373] Demographic and Health Surveys 2004. Demographic and Health Surveys Homepage. Available: http://www.measuredhs.com [accessed 15 August 2004].

[b10-ehp0114-000373] Dennis RJ, Maldonado D, Norman S, Baena E, Martinez G (1996). Woodsmoke exposure and risk for obstructive airways disease among women. Chest.

[b11-ehp0114-000373] Edwards R, Smith KR, Zhang J, Ma Y (2004). Implications of changes in household stoves and fuel use in China. Energ Policy.

[b12-ehp0114-000373] Energy Sector Management Assistance Programme 1996. Energy for Rural Development in China: An Assessment Based on a Joint Chinese/ESMAP Study in Six Counties. Washington, DC:The World Bank.

[b13-ehp0114-000373] Energy Sector Management Assistance Programme 2004. The Impact of Energy on Women’s Lives in Rural India. Washington DC:The World Bank.

[b14-ehp0114-000373] Ezzati M, Kammen DM (2001). Quantifying the effect of exposure to indoor air pollution from biomass combustion on acute respiratory infections in developing countries. Environ Health Perspect.

[b15-ehp0114-000373] Intermediate Technology Development Group 2004. Rape and pastoralists conflict. Peace Bulletin 5:7. Available: http://www.itdg.org/?id=peace5_rape [accessed 31 January 2006].

[b16-ehp0114-000373] IEA and OECD 2004. World Energy Outlook 2004. Paris:International Energy Agency and Organisation for Economic Co-operation and Development.

[b17-ehp0114-000373] Johnson AW, Aderele WI (1992). The association of household pollutants and socio-economic risk factors with the short-term outcome of acute lower respiratory infections in hospitalized pre-school Nigerian children. Ann Trop Paediatr.

[b18-ehp0114-000373] Liu Q, Sasco AJ, Riboli E, Hu MX (1993). Indoor air pollution and lung cancer in Guangzhou, People’s Republic of China. Am J Epidemiol.

[b19-ehp0114-000373] Mavlankar DV, Trivedi CR, Gray RH (1991). Levels and risk factors for perinatal mortality in Ahmedabad, India. Bull WHO.

[b20-ehp0114-000373] Mehta S Gore F Prüss-Üstün Rehfuess E Smith K In press. Modeling household solid fuel use towards reporting of the Millennium Development Goal indicator. Energy for Sustainable Development.

[b21-ehp0114-000373] Mehta S Shapar C 2004 The health benefits of interventions to reduce indoor air pollution from solid fuel use. A cost-effectiveness analysis Energy for Sustainable Development 8 3 53 59Available: http://ieiglobal.org/ESDVol8No3/cost-effectiveness.pdf [accessed 31 January 2006].

[b22-ehp0114-000373] Mishra V, Dai X, Smith KR, Mika L (2004). Maternal exposure to biomass smoke and reduced birth weight in Zimbabwe. Ann Epidemiol.

[b23-ehp0114-000373] Mishra V Retherford RD Smith KR 2005. Cooking smoke and tobacco smoke as risk factors for stillbirth. Int J Environ Health Res 15(6). 10.1080/09603120500288913 16506434

[b24-ehp0114-000373] Modi V McDade S Lallement D Saghir J 2005. Energy and the Millennium Development Goals. New York:Energy Sector Management Assistance Programme, United Nations Development Programme, UN Millennium Project, World Bank.

[b25-ehp0114-000373] O’Dempsey T, McArdle TF, Morris J (1996). A study of risk factors for pneumococcal disease among children in a rural area of West Africa. Int J Epidemiol.

[b26-ehp0114-000373] Onuba O, Udoidiok E (1987). The problems of burns and prevention of burns in developing countries. Burns.

[b27-ehp0114-000373] Pandey MR (1984). Domestic smoke pollution and chronic bronchitis in a rural community of the hill region of Nepal. Thorax.

[b28-ehp0114-000373] Pandey MR (1997). Women, wood energy and health. Wood Energy News.

[b29-ehp0114-000373] Pandey MR, Neupane RP, Gautam A, Shrestha I (1989). Domestic smoke pollution and acute respiratory infections in a rural community of the hill region of Nepal. Environ Int.

[b30-ehp0114-000373] Perez-Padilla R, Regalado J, Vedal S (1996). Exposure to biomass smoke and chronic airways disease in Mexican women: a case-control study. Am J Respir Crit Care.

[b31-ehp0114-000373] Pokhrel AK, Smith KR, Khalakdina A, Deuja A, Bates MN (2005). Case-control study of indoor cooking smoke exposure and cataract in Nepal and India. Int J Epidemiol.

[b32-ehp0114-000373] Robin LF, Less PS, Winget M (1996). Wood-burning stoves and lower respiratory illness in Navajo children. Pediatr Infect Dis J.

[b33-ehp0114-000373] Sinton JE, Smith KR, Peabody J, Yaping L, Ziliang Z, Edwards R (2004). An assessment of programs to promote improved household stoves in China. Energy for Sustainable Development.

[b34-ehp0114-000373] Smith KR 1987. Biofuels, Air Pollution and Health: A Global Review. New York:Plenum Press.

[b35-ehp0114-000373] Smith KR Mehta S Maeusezahl-Feuz M 2004. Indoor air pollution from household use of solid fuels. In: Comparative Quantification of Health Risks: Global and Regional Burden of Disease Attributable to Selected Major Risk Factors (Ezzati M, Lopez AD, Rodgers A, Murray CJL, eds). Geneva:World Health Organization, 1435–1493. Available: http://www.who.int/publications/cra/chapters/volume2/1435-1494.pdf [accessed 31 January 2006].

[b36-ehp0114-000373] Smith KR, Samet JM, Romieu I, Bruce N (2000a). Indoor air pollution in developing countries and acute lower respiratory infections in children. Thorax.

[b37-ehp0114-000373] Smith KR, Uma R, Kishore VVN, Zhang J, Joshi V, Khalil MAK (2000b). Greenhouse implications of household stoves: an analysis for India. Annu Rev Energ Env.

[b38-ehp0114-000373] United Nations 2005a. Millennium Development Goals. Available: http://www.un.org/millenniumgoals/[accessed24 April 2005].

[b39-ehp0114-000373] United Nations 2005b. Proportion of Population Using Solid Fuels. Millennium Development Goals Indicator Database, Goal 7, Indicator 29 New York:United Nations Statistics Division. Available: http://millenniumindicators.un.org/unsd/mi/mi_goals.asp [accessed 24 April 2005].

[b40-ehp0114-000373] United Nations 2000. United Nations Millennium Declaration. Available: http://www.un.org/millennium/declaration/ares552e.pdf [accessed 24 April 2005].

[b41-ehp0114-000373] United Nations High Commissioner for Refugees 2001. Evaluation of the Dadaab Firewood Project, Kenya. Geneva:United Nations High Commissioner for Refugees.

[b42-ehp0114-000373] United Nations Millennium Project 2005. Investing in Development: A Practical Plan to Achieve the Millennium Development Goals. London:Earthscan. Available: http://www.unmillenniumproject.org/reports/fullreport.htm [accessed 31 January 2006].

[b43-ehp0114-000373] U.S. EPA 1996. Air Quality Criteria for Particulate Matter. Vol 1. EPA/600/P-95/001.aF. Research Triangle Park, NC:U.S. Environmental Protection Agency.

[b44-ehp0114-000373] Wang TJ, Zhou BS, Shi JP (1996). Lung cancer in nonsmoking Chinese women: a case-control study. Lung Cancer.

[b45-ehp0114-000373] WHO 2002. World Health Report 2002: Reducing Risks, Promoting Healthy Life. Geneva:World Health Organization. Available: http://www.who.int/whr/2002/en/[accessed 31 January 2006].

[b46-ehp0114-000373] WHO 2005a. World Health Statistics 2005. Geneva:World Health Organization. Available: http://www.who.int/healthinfo/statistics/en/index.html [accessed 31 January 2006].

[b47-ehp0114-000373] WHO 2005b. World Health Survey. Geneva:World Health Organization. Available: http://www.who.int/whs/[accessed 24 April 2005].

[b48-ehp0114-000373] WHO 2005c. Global Indoor Air Pollution Database. Geneva:World Health Organization. Available: http://www.who.int/indoorair/health_impacts/databases_iap/[accessed 5 April 2005].

[b49-ehp0114-000373] WHO 2005d. World Health Report 2005: Make Every Mother and Child Count. Geneva:World Health Organization. Available: http://www.who.int/whr/2005/whr2005_en.pdf [accessed 31 January 2006].

[b50-ehp0114-000373] WHO 2005e. Indoor Air Thematic Briefing 2: Indoor Air Pollution, Health and the Burden of Disease. Geneva:World Health Organization. Available: http://www.who.int/indoorair/info/briefing2.pdf [accessed 31 January 2006].

[b51-ehp0114-000373] World Bank 2006. Living Standards Measurement Study. Available: http://www.worldbank.org/lsms/[accessed on 1 February 2006].

[b52-ehp0114-000373] Wu-Williams AH, Dai XD, Blot W, Xu ZY, Sun XW, Xiao HP (1990). Lung cancer among women in north-east China. Br J Cancer.

